# Oncologic Outcomes of Breast-Conserving Surgery in a Colombian Cancer Center: An Observational, Analytical, Retrospective Cohort Study

**DOI:** 10.3390/cancers17071131

**Published:** 2025-03-28

**Authors:** Sandra E. Díaz-Casas, Flavio J. Rosero-Díazdel Castillo, Sara Mendoza-Díaz, Andersson Sáenz-Ladino, Ricardo Sánchez-Pedraza, Sonia P. Silva-Cárdenas, Andrea Zuluaga-Liberato, Ximena Briceño-Morales, Luis Guzmán-AbiSaab, Óscar Gamboa-Garay, Javier Ángel-Aristizábal, Iván Mariño-Lozano, Raúl Suárez-Rodríguez, Mauricio García-Mora, Carlos Duarte-Torres, Marcela Núñez-Lemus

**Affiliations:** 1 Functional Unit for Breast and Soft Tissue Tumors, Instituto Nacional de Cancerología, Bogotá 111511, Colombia; 2 Fundación Universitaria de Ciencias de la Salud, Bogotá 111411, Colombia; 3 Radiation Oncology Area Group, Instituto Nacional de Cancerología, Bogotá 111511, Colombia; 4 Universidad Nacional de Colombia, Bogotá 111321, Colombia; 5 Clinical Oncology Functional Unit, Instituto Nacional de Cancerología, Bogotá 111511, Colombia; 6 Office of the Deputy Director of Research, Epidemiological Surveillance, Promotion, and Prevention of Cancer, Instituto Nacional de Cancerología, Bogotá 111321, Colombia

**Keywords:** breast neoplasms, mastectomy, segmental, radiotherapy, adjuvant, neoplasm recurrence, local, disease-free survival

## Abstract

**Background:** Breast-conserving surgery (BCS) is one of the major surgical advances in breast cancer treatment. This study evaluated the oncological outcomes of BCS in patients with non-metastatic breast cancer at a referral cancer center in a medium-resource country between 2013 and 2019. **Methods:** An observational, analytical, retrospective cohort study was conducted on patients with stage I–IIIC breast cancer treated at the Instituto Nacional de Cancerología (Bogotá, Colombia) from September 2013 to March 2019. Demographic data, tumor characteristics, treatment types, and survival outcomes were retrospectively collected. **Results:** A total of 409 patients were included. In 64.1% of cases, BCS was performed as the initial treatment and in 35.9%, after neoadjuvant chemotherapy (NACT). With a median follow-up of 85.2 months, tumor recurrence was documented in 9.04% of patients, local recurrence in 2.9%, regional in 2.2%, and distant in 5.6%. The identified risk factors for mortality were a locally advanced clinical stage (HR 5.13; *p* = 0.01), triple-negative subtype (HR 8.02; *p* < 0.01), and nodal involvement of more than four lymph nodes in the surgical specimen (HR 4.00; *p* < 0.01). **Conclusions:** Breast-conserving surgery is an oncologically safe procedure for patients with early and locally advanced breast cancer who respond to NACT. The time to recurrence and overall survival are determined by the clinical stage, axillary tumor burden, and biological subtype of the disease.

## Simple Summary

This study was carried out at the National Cancer Institute, in Colombia, which is a national reference center, where most of the patients arrive in locally advanced stages. We show that breast-conserving surgery is an oncological safe procedure for patients with early and locally advanced breast cancer, who respond to neoadjuvant chemotherapy. Oncological outcomes, like the time to recurrence and overall survival, are determined by clinical stage, axillary tumor burden, and biological subtype of the disease.

## 1. Introduction

Breast cancer is the most commonly diagnosed neoplasm worldwide, regardless of the degree of national development. According to GLOBOCAN reports for 2022, the estimated number of new cases worldwide is 2,296,840, with an incidence rate of 46.8 cases and a mortality rate of 12.7 per 100,000 [1]. In women, it is the most common cancer in terms of incidence and the leading cause of mortality worldwide. In Colombia, an estimated 17,018 new cases were reported in 2022, with an incidence rate of 50.7 and a mortality rate of 13.3/100,000. This represents 27.7% of all neoplasms in Colombian women, with the highest incidence and mortality [2]. Breast cancer management requires a multidisciplinary approach that includes surgery, radiotherapy, and systemic treatment with endocrine therapy, chemotherapy, and/or targeted therapies [3]. Surgical management, as the mainstay of locoregional control, is usually the initial approach for early and locally advanced tumors after neoadjuvant systemic therapy. The surgical treatment of breast cancer evolved from Halsted’s radical mastectomy in 1907 [4] to less aggressive approaches, reaching breast-conserving surgery (BCS), which was established as the standard of management based on studies published in the 1980s by Veronesi with MILAN I, II, and III studies [5,6,7] and the NSABP group study [8]. These clinical trials established the equivalence of BCS plus radiotherapy versus radical surgery in terms of overall survival (OS), with less morbidity and a marked improvement in cosmetic outcomes. This study evaluated the oncologic outcomes of BCS at the Instituto Nacional de Cancerología (INC) (Bogotá, Colombia) between 2013 and 2019 in patients with early and locally advanced tumors following neoadjuvant chemotherapy (NACT), with a minimum follow-up of 5 years.

## 2. Materials and Methods

### 2.1. Study Design and Patient Eligibility

An observational, analytical, historical cohort-type study was developed, which included patients with a confirmed diagnosis of non-metastatic breast cancer registered in the database of the Functional Unit for Breast and Soft Tissue Tumors of the INC (hereinafter, Functional Unit) from 1 September 2013, to 1 March 2019, who underwent BCS and adjuvant radiotherapy (RT).

A search of the database of the Functional Unit located the clinical records of patients who underwent BCS during the period described. Subsequently, these records were reviewed in the SAP medical records system to identify patients who met the following inclusion criteria: histopathological confirmation of infiltrating breast cancer; clinical staging (I to IIIC); and complete treatment with surgery, RT, and systemic therapy at the INC. The study variables included the sociodemographic characteristics of the patients, clinical and anatomic pathology data from the initial biopsy and surgical specimen, types of treatment administered, recurrence sites and dates with treatments for recurrence, and mortality. Three authors extracted and entered the data into an electronic platform designed to manage clinical study information (REDCap). Information quality and fidelity were reviewed by a research assistant assigned to the Research Division of INC. The study was approved by the Ethics Committee of the INC, according to Minutes No. 0017-20 (date 8 July 2020).

### 2.2. Statistical Analysis

All statistical analyses were performed using R-Project software, version 4.3.3 (R Foundation for Statistical Computing, Vienna, Austria). The mean and standard deviation were used for quantitative variables (after validating the assumption of normality using the Kolmogorov–Smirnov test). Qualitative variables were described using absolute and relative frequencies. Outcomes of interest were defined as follows: OS, calculated as the time elapsed between the date of the bi-disciplinary Oncology and Breast Surgery consultation and the patient’s date of death; time to recurrence (TR) (local, regional, or distant), calculated as the time elapsed between the date of the bi-disciplinary Oncology and Breast Surgery consultation and the date of diagnosis of disease recurrence in the operated breast, ipsilateral lymph node chains, or the presence of distant metastases, according to STEEP criteria [9].

The frequency of these outcomes was calculated using incidence rates expressed as events per 100 patients/year, together with their 95% confidence intervals (CIs). Additionally, the percentages of local, regional, and distant recurrences were calculated using the total number of patients in the cohort as the denominator. Cases considered lost to follow-up or not presenting the event of interest were handled as right censoring at the last contact with the INC.

Survival functions were obtained using the Kaplan–Meier estimator and are presented in graphs. The log-rank test was used to evaluate the differences in survival functions. Univariate Cox regression analysis was performed to identify possible risk factors associated with the outcomes of interest. Subsequently, multivariable Cox proportional hazards models were fitted for each outcome to analyze the association between risk factors, including those with a *p*-value < 0.1 from the univariate analysis. The proportional hazards assumption was verified using Schoenfeld residuals to test the hypothesis of a zero slope. Confidence intervals were calculated at 95%. All contrasts were bilateral, with a *p*-value of <0.05, which was considered statistically significant.

## 3. Results

**Figure 1:**
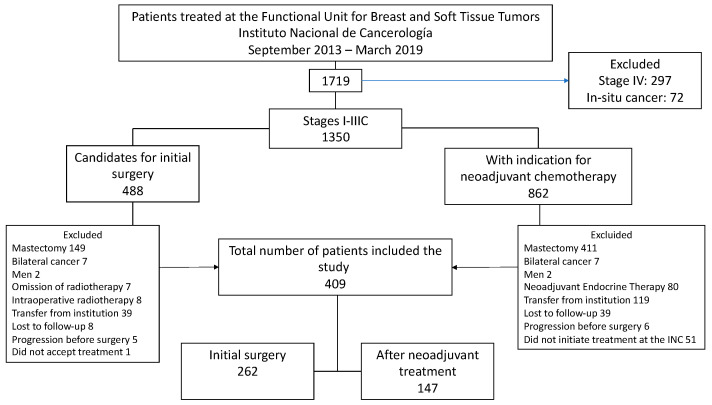
Flowchart of patient selection.

Between 1 September 2013 and 1 March 2019, 1719 patients were seen at the Functional Unit of the INC, and 78.5% (n = 1350) of these corresponded to stage I to IIIC patients. Of this group, 35.9% (n = 488) underwent initial surgery, and 64.1% (n = 862) received NACT. After a thorough review of clinical records, 409 patients met the inclusion criteria for the study. Figure 1 illustrates the causes of exclusion in the final analysis.

The mean age at diagnosis was 58.7; 68.7% (n = 281) of patients had early tumors (stage I–IIA), and 31.3% (n = 128) had locally advanced tumors (IIB-IIIC). The most frequent biological subtype was luminal A (45.2%), with lower representation of aggressive subtypes, such as HER2-positive (15.2%) and triple-negative (8.1%) (Table 1).

A total of 35.9% (n = 147) of patients received NACT, with AC-T (adriamycin and cyclophosphamide followed by taxanes) being the most commonly used regimen in 59.9% (n = 88). A pathologic complete response (pCR) was achieved in 25.2% (n = 37) of the cases. All patients in the cohort underwent quadrantectomy; in 16 patients, this procedure was performed within oncological mammoplasty. Of the total number of patients in the cohort, 62.5% (n = 256) underwent a sentinel lymph node biopsy. The sentinel node result was positive for metastases in 30.5% (n = 78) of patients who underwent surgery as the initial treatment. Axillary dissection was omitted in 44.9% (n = 35). In the group of patients who received NACT, sentinel node biopsy was performed in 18 patients (12.2%), with positive results for metastasis in two patients (11.1%) who underwent axillary lymphadenectomy. Positive resection margins were reported in 12.7% (n = 52) of patients, with only one involved margin in 84.6% (n = 44) of the cases. In patients with positive margins, margin enlargement was performed in 65.4% (n = 34), simple mastectomy in 7.7% (n = 4), and RT was the only treatment in 26.9% (n = 14), highlighting that in this group, node involvement was of only one margin. Of the 38 patients who underwent margin enlargement or simple mastectomy, only 17 had residual tumors (11 with infiltrating tumors and 6 with in situ involvement). Two of the 11 patients with residual infiltrating tumors had positive margins again and were referred for mastectomy (Table 1 and Table 2).

Regarding systemic adjuvant treatment, 38.1% (n = 156) of the patients required adjuvant chemotherapy with or without targeted therapy, 89.2% (n = 365) received endocrine therapy, and 99.5% (n = 407) received adjuvant RT; 3D-CRT was used in 90.9% (n = 370) of the cases. Two patients did not receive RT because they had positive margins, underwent simple mastectomy, and did not receive any indication for adjuvant RT.

### 3.1. Local, Distant, Regional, or Mixed Recurrence

Of the 409 patients, 92.7% (n = 377) were alive and disease-free at 60 months. The median follow-up time was 85.2 months (interquartile range [IQR]: 65.9;99.9), with a minimum of 4.40 months and a maximum of 116.5 months. During follow-up, 37 patients (9.04%) experienced distant recurrence, locoregional recurrence, or both, with distant being the most frequent site (n = 23, 5.62%), followed by local recurrence (n = 12, 2.93%) in the same quadrant (in 10 of these cases), and regional recurrence (n = 9, 2.2%). Additionally, six of these patients presented with mixed recurrence, with local and distant combination being the most frequent (n = 3); two patients presented with regional and distant recurrence, and one patient progressed locally, regionally, and distantly.

The most frequent site of systemic recurrence was the bone in 12 of the 23 patients, which was related to the dissemination pattern of the luminal tumors. When analyzing the patients with local recurrence, it was found that all 12 patients had negative resection margins in the surgical specimen, half of them had early tumors, 10 received reinforcement with RT in the surgical bed (6 patients were <60 years old and 4 had histologic grade III tumors), and 7 of them received NACT.

Among patients with regional recurrence, six of nine had early tumors. In five of them, a sentinel lymph node biopsy was performed, which was positive for metastasis in four patients, fulfilling the criteria for omission of axillary dissection in only one patient. Four of the nine patients with regional recurrence had luminal B tumors (three were HER2-negative), three patients also had distant recurrence, and of the four patients who had undergone axillary dissection, two had more than four positive lymph nodes. In other words, of the 35 patients for whom lymphadenectomy was omitted with a positive sentinel lymph node, only one had regional recurrence at the level of the supraclavicular fossa. Of the nine regional recurrences, five occurred in the supraclavicular fossa, three in the ipsilateral axilla, and one in the infraclavicular fossa.

Regarding radiotherapy, of the nine patients with regional recurrence, four had more than four positive lymph nodes on surgical pathology. All patients underwent axillary dissection; however, only one patient received axillary nodal RT. Most patients with distant recurrence (12 of 23) had locally advanced tumors, and 13 of them had luminal B tumors (2 were HER2-positive).

When analyzing the 32 (7.82%) patients with clinical stage IIIB, we found that only 2 had local recurrence, and 1 of them had distant recurrence but died from COVID-19. Of the 26 patients with multifocal tumors, 6 presented recurrences (2 local, 2 regional, and 2 distant), and 4 of them died as a consequence of the disease.

Regarding systemic treatment, of the 37 patients with recurrence, 22 received NACT: 4 with pCR (one triple-negative and three HER2-positive) and 18 with residual disease (10 with luminal B HER2-negative tumors). All patients with distant recurrence received neoadjuvant regimens according to biological subtype, the most frequent being AC-T. In addition, all patients with positive hormone receptor expression (n = 28) received adjuvant endocrine therapy, with tamoxifen being the most frequent. Most of the 23 patients with distant recurrence (including mixed recurrence) had luminal B HER2-negative tumors (n = 15), 3 had triple-negative cancer, 3 were HER2-positive, and 2 patients had luminal A tumors. As systemic treatment for distant recurrence, 5 patients received chemotherapy, 8 underwent endocrine therapy, and 10 received endocrine therapy + iCDK4/6. In the last review of the 23 patients with systemic recurrence, 20 died and 3 remained alive under treatment for their disease (Table 3).

### 3.2. Univariate and Multivariate Analysis of Recurrences

**Figure 2:**
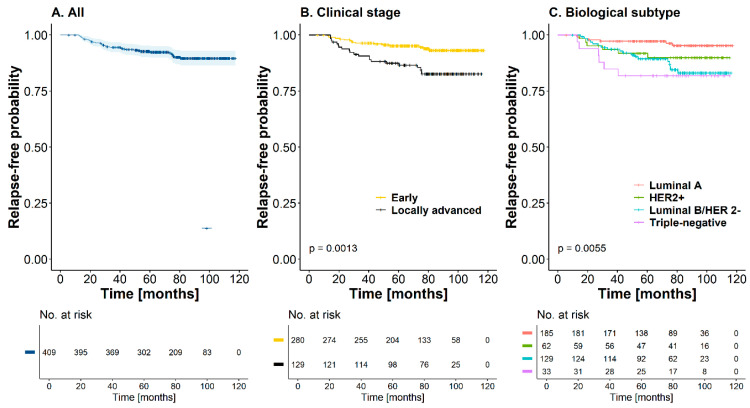

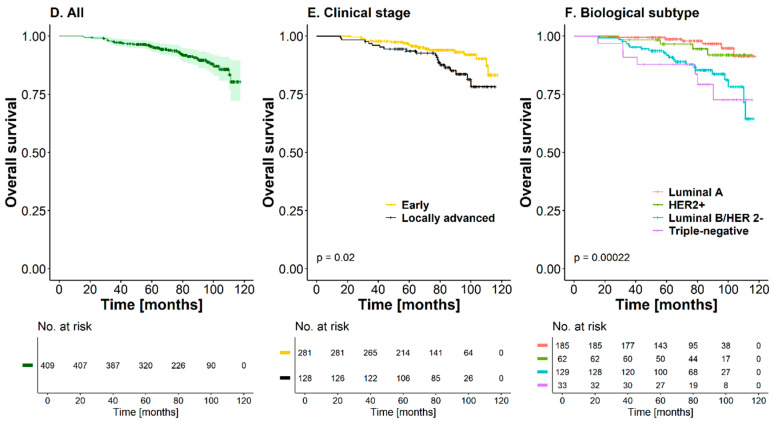
Time to recurrence and overall survival (Kaplan–Meier curves) in breast cancer patients in relation to the whole cohort, clinical stage, and biological subtype.

At 60 months, survival was 92.4% (CI95%: [89.9; 95.1]) for the entire cutoff without reaching the median (Figure 2A). In Kaplan–Meier curves for the recurrence-free time, a statistically significant difference was found according to the stage and biological subtype, with worse prognosis in patients with locally advanced tumors (HR = 2.78, CI95%: [1.47; 5.38], *p*-value < 0.01) and a triple-negative subtype (HR = 4.99, CI95%: [1.68; 14.4], *p*-value < 0.01) (Figure 2B,C) (Table 4). The recurrence incidence rate was 1.49 recurrence events per 100 patients/year (CI95%: [1.05, 2.05]). However, the relationship between the possible prognostic factors and recurrence was evaluated. Unadjusted, five variables were found to be associated with a worse prognosis regarding the time to recurrence (of any type): clinical stage, biological subtype, number of involved lymph nodes, tumor extension, and primary treatment. However, on an adjusted basis, taking patients with luminal A tumors as a reference, the probability of recurrence was higher in those patients with luminal B HER2-negative tumors (HR = 3.06, CI95%: [1.24; 7.56], *p*-value = 0.016) or triple-negative tumors (HR = 4.46, CI95%: [1.37; 14.6], *p*-value = 0.013). Compared with patients who did not have involved lymph nodes, the risk of recurrence was higher in those cases with more than four lymph nodes (HR = 2.56, CI95%: [1.08; 6.06], *p*-value = 0.033). Finally, multifocal tumors had an increased risk of recurrence that was statistically significant compared to unifocal tumors (HR = 3.31, CI95%: [1.37; 8.04], *p*-value < 0.01). Although not statistically significant, the risk of recurrence was higher in patients who received NACT as an initial treatment than in those who underwent surgery for primary tumor management (Table 4). On the other hand, when variables related to local recurrence were analyzed using the Cox proportional hazards model, it was found that the triple-negative biological subtype was highly associated with this type of recurrence (HR = 8.34, CI95%: [1.39; 50], *p*-value = 0.021). At the same time, In patients with distant recurrence, the luminal B HER2-negative biological subtype (HR = 8.07, CI95%: [1.80; 36.1], *p*-value < 0.01) and locally advanced tumors (HR = 3.26, CI95%: [1.01; 10.6], *p*-value = 0.049) had a statistically significant asso ciation with a worse prognostic trend regarding this outcome.

### 3.3. Overall Survival

During the entire follow-up, a total of 38 deaths (9.2%) occurred; 27 of these patients had T2 tumors, half of them (n = 19) were node-positive, and 21 had luminal B HER2-negative tumors. At 60 months, the OS rate was 95.4% (CI95%: [93.3; 97.5]) for the entire cohort; the median OS was not reached for this cohort of patients (Figure 2D). The mortality rate was 1.39 deaths per 100 patient-years (CI95%: [0.98; 1.91]). Similar to the recurrence-free time outcome, a statistically significant difference was found between curves according to the clinical stage and biological subtype, with a worse prognosis in patients with locally advanced tumors (HR = 2.09, CI95%: [1.11; 3.92], *p*-value = 0.023) and with triple-negative biological subtypes (HR = 6.13, CI95%: [2.05; 18.3], *p*-value < 0.01) (Figure 2E,F). For this outcome, the adjusted analysis showed that a locally advanced clinical stage (HR = 5.13, CI95%: [1.49; 17.6], *p*-value < 0.01), triple-negative subtype (HR = 8.02, CI95%: [1.79; 35.9], *p*-value < 0.01), and nodal involvement greater than four lymph nodes in the surgical specimen (HR = 4.00, CI95%: [1.44; 11.3], *p*-value < 0.01) were statistically significant factors that determined a worse prognosis in this cohort of patients with OS. The primary treatment did not have an important effect on this outcome (HR = 1.22, CI95%: [0.36; 4.18], *p*-value = 0.750) when adjusted for the other covariates (Table 4).

## 4. Discussion

This cohort describes our experience in the management of breast cancer using breast-conserving surgery (BCS) in a reference cancer center in Colombia, with a median follow-up of 85 months. Patients undergoing initial surgery were included (64.1%, n = 262), of which 243 (92.7%) had early tumors. Patients undergoing BCS following neoadjuvant chemotherapy were also included (35.9%, n = 147), of which 110 (74.8%) had locally advanced tumors.

In the entire cohort, a local recurrence rate of 2.93% was found, which is a lower percentage than that reported in the studies that established the safety of quadrantectomy plus RT compared to radical surgery [5,6,7,8,10,11,12,13,14,15]. However, it is important to note that these studies included only patients with T1-T2 and N0 tumors. For BCS, in Fisher’s study (NSABP B-06) [8], the local recurrence rate was 14.3%. In MILAN I [5], it was 8.8% in patients with tumors smaller than 2 cm; similarly, MILAN III [7], which evaluated the addition of radiotherapy to BCS in tumors <2.5 cm, found recurrence rates of 0.3% with RT vs. 8.8% without it. Finally, the Early Breast Cancer Trialists’ Collaborative Group (EBCTCG) meta-analysis, which included 10,801 patients, reported a local recurrence rate of 8% [14].

In the INC cohort, resection border involvement was present in 12.7% (n = 52) of patients, without this being associated with local recurrence, TR, or OS. Notably, the highest percentage of positive margins occurred in patients who underwent surgery as the initial treatment. In the European Organisation for Research and Treatment of Cancer (EORTC) 10801 trial [15], it was found that although BCS has a higher risk of recurrence at 10 years (19.7% vs. 12.2% with mastectomy), when the status of the margins was also considered, recurrence rates decreased to 17.6% in the case of complete resection vs. no resection (26.5%). Compared with reports worldwide, the rate of positive margins at the INC is well below the 21.6% rate reported for the United States according to the Mastery of Breast Surgery program of the American Society of Breast Surgeons [16].

During the time of the study, of the group of patients with locally advanced tumors who received NACT with complete oncologic treatment at the INC (n = 558), 411 had mastectomy, and 147 patients underwent BCS. In other words, a BCS rate of 26.3% was achieved, a percentage similar to that in the NSABP B-27 study [17], which used the AC-T scheme and achieved a BCS rate of 24.6%. This suggests the possibility of de-escalating surgical management and preserving the breast in almost one-third of patients with locally advanced tumors, which reduces the surgical time and morbidity, improves aesthetic results, and improves the quality of life of patients [18,19]. In the EORTC 10902 study [20], the BCS rate following NACT was 35%, with 29% of participants having T3–T4 tumors and 50% having positive lymph nodes. In the present study, 4.9% (n = 20) of patients had T3 tumors, 7.8% (n = 32) were T4b, and 30.5% were lymph node-positive, a relatively similar population to the previously mentioned study. A previous publication by the INC Functional Unit reported a BCS rate of 36.7% in locally advanced tumors that had received NACT [21].

Patients who achieved a significant decrease in tumor volume with NACT and underwent BCS had the same oncologic outcomes as those who underwent initial surgery. This was corroborated in the study of Cho et al. [22] based on 124 patients with locally advanced tumors who received NACT, with local recurrence rates of 5% at 5 years. Shin et al. [23] reported recurrence-free survival rates of 90.9% in patients with locally advanced tumors who underwent BCS after NACT, with no statistically significant differences when compared to mastectomy. Furthermore, in a study by Gwark et al. [24], BCS was significantly associated with better survival outcomes. Hage et al. [25] concluded that the BCS rate is a better measure of the impact of NACT than the pathologic complete response (pCR) itself.

In this cohort, pCR was 25.2%, very similar to that reported in the NSABP B-27 study [17] and to that previously described by the INC’s Functional Unit [21], in which 52.2% of the patients had luminal tumors (A and B HER2-negative) with a pCR of 10.6% for this biological subtype, higher than that reported by Hage et al. [25] with a pCR of 6% in this group. When analyzing HER2-positive tumors that received neoadjuvant therapy with AC-TH in this cohort, pCR was 37.2%, similar to that reported in the NOAH study [26] with 38% and higher than the NeoALTTO study [27] with 27.6%. For the triple-negative subtype, AC-T was the most commonly used scheme, with a pCR of 44% reported, lower than the clinical trials GeparSixto [28] with 53%, CALGB 40603 [29] with 54%, and BrighTNess [30] with 58%. This may be explained by the fact that only seven patients received carboplatin added to the taxane phase. The use of platinum is not yet a standard therapy in patients with triple-negative tumors, nor is dual anti-HER2 blockade used in patients with HER2-positive tumors. Immunotherapy was approved by the regulatory entity of Colombia (Invima) in August 2024. Currently, dual anti-HER2 blockade improves pCR rates between 45% and 75%, as evidenced by the following trials: NeoSphere [31], 39.3%; BERENICE [32], 61.8%; TRYPHAENA [33], 66.2%; and TRAIN-2 [34], 68%. Similarly, for the triple-negative subtype, the addition of immunotherapy achieved a pCR rate of 64.8% [35].

In this study, the 5-year OS rate was 95.4%, similar to that reported in the EBCTCG meta-analysis [14], which reported a 5-year cancer mortality of 6.8% (OS 93.2%) and below the all-cause death rate in the same follow-up period, which was 10.3%. Corradini et al. [36] reported data from the Munich Cancer Registry (in 7500 patients) and found a 5-year OS of 93.8% for those who underwent BCS. In a study by Almahariq et al. [37], a matched cohort analysis of patients with early tumors (T1–2, N0) undergoing BCS was performed, and the 5-year OS was 94.4%. Finally, in the Sun et al. study [38], which included 4262 patients, the OS was 96.5% for patients undergoing BCS. All three studies were conducted based on cohorts of patients with early-stage tumors. The above data show concordance between the outcomes of the present cohort and the literature, both in developed and developing countries.

Finally, in this cohort, the factors associated with a lower OS were locally advanced clinical stages, a triple-negative biological subtype, and lymph node involvement greater than four in the surgical specimen, which are widely known prognostic factors associated with worse outcomes in breast cancer [39]. Therefore, it should be highlighted that clinical studies that established the safety of BCS did not have information regarding the tumor biology, and current evidence shows that the clinical stage and tumor biology are the main determinants of oncologic outcomes in breast cancer patients.

The limitations of this study include its retrospective nature and the lack of follow-up in patients with luminal tumors, considering that recurrence in these tumors mainly occurs late and that with new systemic therapies, a higher percentage of BCS will likely be achieved in locally advanced tumors with triple-negative and HER2 biologic subtypes. The strength of this study results from its sample size with a large number of patients with locally advanced tumors from a national reference cancer center.

## 5. Conclusions

Breast-conserving surgery at the Instituto Nacional de Cancerología (Bogotá, Colombia) is an oncologically safe procedure for patients with early and locally advanced breast cancer who respond to neoadjuvant chemotherapy, with a significant reduction in tumor volume. The time to recurrence and overall survival are determined by the clinical stage, axillary tumor burden, and biological subtype of the disease.

## Figures and Tables

**Table 1:** Main characteristics of patients diagnosed with non-metastatic breast cancer included in the cohort (n = 409).

Clinical and Histopathological Data		
Age (Years)	Mean ± SD	58.7 ± 10.7
Tumor size (T), n (%)	T1	127 (31.3)
	T2	230 (56.2)
	T3	20 (4.90)
	T4b	32 (7.80)
Node involvement (N), n (%)	N0	284 (69.4)
	N1	86 (21.0)
	N2a	37 (9.00)
	N2b	1 (0.24)
	N3c	1 (0.24)
Clinical stage, n (%)	I	115 (28.1)
	IIA	166 (40.6)
	IIB	60 (14.7)
	IIIA	36 (8.80)
	IIIB	31 (7.60)
	IIIC	1 (0.24)
Histological type ^†^, n (%)	Ductal (NOS)	362 (88.5)
	Lobular	18 (4.43)
	Medullary	1 (0.24)
	Mucinous	8 (1.97)
	Papillary	5 (1.20)
	Tubular	12 (2.90)
	Other	3 (0.73)
Histological grade, n (%)	I	70 (17.1)
	II	265 (64.8)
	III	74 (18.1)
Molecular subtype, n (%)	Luminal A	185 (45.2)
	Luminal B HER2-positive	53 (13.0)
	Luminal B HER2-negative	129 (31.5)
	Pure HER	9 (2.20)
	Triple-negative	33 (8.10)
Prognostic data of the surgical specimen		
Tumor extension ^§^, n (%)		
	Unifocal	383 (93.6)
	Multifocal	26 (6.4)
Sentinel lymph node report ^§^, n (%)		256 (62.6)
*Sentinel lymph node result*	Negative	*178 (69.5)*
	Positive	*78 (30.5)*
	Macrometastasis	*54 (69.2)*
	Micrometastasis	*21 (26.9)*
	Isolated tumor cells	*3 (3.80)*
*Number of positive lymph nodes*	1	*55 (70.5)*
	[2,3]	*20 (25.6)*
	≥4	*3 (3.80)*
Number of lymph nodes at axillary dissection ^§^		
	0	264 (64.5)
	[1–3]	102 (24.9)
	≥4	43 (10.5)
Positive resection margins ^¶^, n (%)		52 (12.7)
*Resection margin status*	1 margin	*44 (84.6)*
	2 margins	*6 (11.5)*
	3 margins or more	*2 (3.80)*
Pathological response *, n (%)		147 (35.9)
	Class 1	*37 (25.2)*
	Class 2	*16 (10.9)*
	Class 3	*88 (59.9)*
	Class 4	*6 (4.10)*

**Abbreviations:** SD: standard deviation; ^†^: in biopsy; ^§^: percentage over the entire cohort of patients (N = 409); ǂ: percentage over those patients with positive sentinel lymph node report; ^¶^: percentage over those patients with positive resection margins; *: percentage over those patients who underwent neoadjuvant chemotherapy.

**Table 2:** Types of treatment administered to patients in the cohort.

Treatment Type ^§^		n (%)
Initial breast-conserving surgery** ^§^**		**262 (64.1)**
*Type of axillary surgery in initial breast-conserving surgery*	*Sentinel lymph node*	238 (90.8)
	*Axillary dissection*	24 (9.20)
Surgery after neoadjuvant chemotherapy ^§^		**147 (35.9)**
*Type of axillary surgery following neoadjuvant chemotherapy*	*Sentinel lymph node*	18 (12.2)
	*Axillary dissection*	129 (87.8)
Neoadjuvant chemotherapy, n (%)		
Chemotherapy regimen		147 (35.9)
	*AC-T*	88 (59.9)
	*AC-TC*	7 (4.80)
	*AC-TH*	38 (25.9)
	*Others*	14 (5.40)
Treatments for positive margins		**52 (12.7)**
	*Simple mastectomy*	4 (7.70)
	*Re-quadrantectomy*	34 (65.4)
	*Radiotherapy*	14 (26.9)
Management of positive sentinel lymph node ^§^		**78 (30.5)**
*Management type*	*Omission of dissection*	35 (44.9)
	*Lymph node dissection*	43 (55.1)
Adjuvant systemic therapy, n (%)		**156 (38.1)**
	*Chemotherapy alone*	92 (58.9)
	*Chemotherapy + target therapy*	28 (17.9)
	*Target therapy alone*	35 (22.4)
	*Other*	1 (0.64)
Adjuvant radiotherapy ^§^		**407 (99.5)**
*Radiotherapy technique used*	*3D-CRT*	370 (90.9)
	*IMRT*	31 (7.60)
	*VMAT*	6 (1.50)
Adjuvant endocrine therapy ^§^		**365 (89.2)**
*Type of endocrine therapy used*	*Tamoxifen*	189 (51.8)
	*Aromatase inhibitor*	105 (28.8)
	*Tamoxifen + aromatase inhibitor*	70 (19.2)
	*GnRH analog + AI*	1 (0.30)

Abbreviations: 3D-CRT: three-dimensional conformal radiotherapy; IMRT: intensity-modulated radiation therapy; VMAT: volumetric intensity-modulated arc therapy; AC: anthracycline, cyclophosphamide; T: taxanes; TH: taxanes and trastuzumab, TC: taxane and cyclophosphamide; AI: aromatase inhibitor. ^§^: Percentage over the entire cohort of patients (N = 409).

**Table 3:** Characterization of tumor recurrences and types of treatment administered in these patients.

Characteristic		Primary Treatment	
	Total	Initial Surgery	Neoadjuvant Chemotherapy
Local	12 (2.93)		
Regional	9 (2.2)		
Distant	23 (5.62)		
**Type of recurrence ^§^**			
Local ^§^, n (%)	12 (27.3)	5 (41.6)	7 (58.3)
Recurrence site			
Same quadrant	10 (83.3)	4 (80.0)	6 (85.7)
Different quadrant	2 (16.7)	1 (20.0)	1 (14.2)
Regional ^§^, n (%)	9 (18.1)	6 (62.5)	3 (37.5)
Distant ^§^, n (%)	23 (52.3)	8 (34.8)	15 (65.2)
Recurrence site			
Bone	12 (52.2)	5 (62.5)	7 (46.6)
Lung	3 (13.0)	1 (12.5)	2 (13.3)
Pleura	3 (13.0)	1 (12.5)	2 (13.3)
Liver	2 (8.70)	0 (0.00)	2 (13.3)
Central nervous system	2 (8.70)	0 (0.00)	2 (13.3)
Non-regional lymph nodes + bone	1 (4.30)	1 (12.5)	0 (0.00)
Treatments used ^†^, n (%)			
Re-quadrantectomy	1 (2.70)	1 (100)	0 (0.00)
Simple mastectomy	8 (21.6)	3 (37.5)	5 (62.5)
Axillary dissection	4 (10.8)	3 (75.0)	1 (25.0)
Chemotherapy	6 (16.2)	2 (33.3)	4 (66.7)
Chemotherapy + target therapy	3 (8.10)	0 (0.00)	3 (100)
Endocrine therapy	15 (40.5)	8 (53.3)	7 (46.7)
Endocrine therapy + target therapy	12 (32.4)	6 (50.0)	6 (50.0)
Radiotherapy	11 (29.7)	3 (27.3)	8 (72.7)
Metastasectomy	3 (8.10)	0 (0.00)	3 (100)
Other	2 (5.40)	0 (0.00)	2 (100)

^†^: Percentage over total number of patients with recurrence (n = 37). ^§^: taking into account that one patient may have had mixed recurrence, the percentage is calculated over the total number of recurrence sites (n = 44).

**Table 4:** Cox proportional hazards model estimates for OS and TR of all types (local, regional, and distant).

	Overall Survival (OS)				Time to Recurrence (TR)			
Characteristic	HR ^a^ [CI95%]	*p*-Value	HR ^b^ [CI95%]	*p*-Value	HR ^a^ [CI95%]	*p*-Value	HR ^b^ [CI95%]	*p*-Value
**Age (years)**								
<50	Ref.				Ref.			
≥50	1.21 [0.55; 3.08]	0.654			0.87 [0.43; 1.91]	0.707		
**Clinical stage**								
Early	Ref.		Ref.		Ref.		Ref.	
Locally advanced	2.09 [1.11; 3.98]	**0.023**	5.13 [1.49; 17.6]	**<0.01**	2.78 [1.47; 5.38]	**<0.01**	2.21 [0.83; 5.90]	0.112
**Tumor size**								
T1-T2	Ref.		Ref.		Ref.		Ref.	
T3-T4	1.53 [0.67; 3.49]	0.106	0.55 [0.13; 2.33]	0.420	1.98 [0.87; 4.06]	0.100	0.76 [0.31; 1.91]	0.566
**Histological grade**								
I-II	Ref.		Ref.		Ref.			
III	2.01 [0.96; 4.20]	0.064	0.74 [0.25; 2.19]	0.590	1.54 [0.70; 3.10]	0.264		
**Biological subtype**								
Luminal A	Ref.		Ref.		Ref.		Ref.	
Her2+	1.72 [0.48; 6.09]	0.401	0.44 [0.05; 4.30]	0.480	2.45 [0.82; 7.08]	0.103	1.78 [0.56; 5.64]	0.330
Luminal B HER2-	4.90 [1.97; 12.1]	**<0.01**	2.97 [0.89; 9.91]	0.077	3.58 [1.59; 8.94]	**<0.01**	3.06 [1.24; 7.56]	**0.016**
Triple-negative	6.13 [2.05; 18.3]	**<0.01**	8.02 [1.79; 35.9]	**<0.01**	4.99 [1.68; 14.4]	**<0.01**	4.46 [1.37; 14.6]	**0.013**
Negative	Ref.		Ref.		Ref.		Ref.	
[1-3]	1.40 [0.64; 3.04]	0.391	0.82 [0.26; 2.58]	0.730	1.19 [0.53; 2.51]	0.651	1.03 [0.44; 2.41]	0.946
≥4	2.25 [0.89; 5.67]	0.086	4.00 [1.44; 11.1]	**<0.01**	2.85 [1.21; 6.13]	**0.018**	2.56 [1.08; 6.06]	**0.033**
**Tumor extensión**								
Unifocal	Ref.				Ref.		Ref.	
Multifocal	2.06 [0.66; 4.96]	0.189			3.44 [1.34; 7.50]	**0.012**	3.31 [1.37; 8.04]	**<0.01**
**Initial treatment**								
Surgery	Ref.		Ref.		Ref.			
Neoadjuvant CT	2.35 [1.18; 4.66]	**0.015**	1.22 [0.36; 4.18]	0.750	2.45 [1.30; 4.81]	**<0.01**	1.13 [0.42; 3.01]	0.810

a: Not adjusted; b: Adjusted; HR: Hazard Ratio; CI: Confidence Interval; CT: Chemotherapy.

## Data Availability

Data are available on request from the authors.

## Abbreviations

The following abbreviations are used in this manuscript:

3D-CRT	Three-dimensional conformal radiotherapy
AC	Anthracycline, cyclophosphamide
AI	Aromatase inhibitor
BCS	Breast-conserving surgery
CI	Confidence intervals
IMRT	Intensity-modulated radiation therapy
INC	Instituto Nacional de Cancerología
IQR	Interquartile range
NACT	Neoadjuvant chemotherapy
OS	Overall survival
pCR	Pathologic complete response
RT	Adjuvant radiotherapy
SD	Standard deviation
T	Taxanes
TC	Taxane and cyclophosphamide
TH	Taxanes and trastuzumab
TR	Time to recurrence
VMAT	Volumetric intensity-modulated arc therapy
